# IoMT-Based Osteosarcoma Cancer Detection in Histopathology Images Using Transfer Learning Empowered with Blockchain, Fog Computing, and Edge Computing

**DOI:** 10.3390/s22145444

**Published:** 2022-07-21

**Authors:** Muhammad Umar Nasir, Safiullah Khan, Shahid Mehmood, Muhammad Adnan Khan, Atta-ur Rahman, Seong Oun Hwang

**Affiliations:** 1 Riphah School of Computing & Innovation, Faculty of Computing, Riphah International University, Lahore Campus, Lahore 54000, Pakistan; m.nasir@riphah.edu.pk (M.U.N.); shahid.mahmood@riphah.edu.pk (S.M.); 2 Department of IT Convergence Engineering, Gachon University, Seongnam 13120, Korea; safi@gachon.ac.kr; 3 Pattern Recognition and Machine Learning Lab., Department of Software, Gachon University, Seongnam 13120, Korea; 4 Department of Computer Science, College of Computer Science and Information Technology, Imam Abdulrahman Bin Faisal University, P.O. Box 1982, Dammam 31441, Saudi Arabia; aaurrahman@iau.edu.sa; 5 Department of Computer Engineering, Gachon University, Seongnam 13120, Korea

**Keywords:** blockchain, fog computing, edge computing, osteosarcoma cancer, transfer learning, IoMT

## Abstract

Bone tumors, such as osteosarcomas, can occur anywhere in the bones, though they usually occur in the extremities of long bones near metaphyseal growth plates. Osteosarcoma is a malignant lesion caused by a malignant osteoid growing from primitive mesenchymal cells. In most cases, osteosarcoma develops as a solitary lesion within the most rapidly growing areas of the long bones in children. The distal femur, proximal tibia, and proximal humerus are the most frequently affected bones, but virtually any bone can be affected. Early detection can reduce mortality rates. Osteosarcoma’s manual detection requires expertise, and it can be tedious. With the assistance of modern technology, medical images can now be analyzed and classified automatically, which enables faster and more efficient data processing. A deep learning-based automatic detection system based on whole slide images (WSIs) is presented in this paper to detect osteosarcoma automatically. Experiments conducted on a large dataset of WSIs yielded up to 99.3% accuracy. This model ensures the privacy and integrity of patient information with the implementation of blockchain technology. Utilizing edge computing and fog computing technologies, the model reduces the load on centralized servers and improves efficiency.

## 1. Introduction

Osteosarcoma is the most common type of cancer that arises in the bones. On the surface, these tumors appear to resemble early types of bone cells that help to forge new bone tissue, but the tissue in osteosarcoma is softer and weaker than normal bone tissue. Adolescents, teenagers, and young adults are the most commonly affected by osteosarcomas. Despite the fact that teenagers are the most usually affected age group, osteosarcoma may strike at any age. In adolescents, teenagers, and young adults, osteosarcoma typically originates in areas where the bone is quickly increasing, such as around the distal area of the limb or arm bones. The majority of tumors grow in the bones around the knee, either in the distal femur (lower thigh bone) or in the proximal tibia (upper shin bone). The upper arm bone near the shoulder (proximal humerus) is the next most commonly affected area. Almost 1000 people are diagnosed with osteosarcoma in the USA each year. Tumors in children and adolescents under the age of 14 are osteosarcomas in 2% of cases, and 3% of tumors in teenagers above the age of 14 are osteosarcomas. Teenage patients account for the majority of cases; most of them are diagnosed between the ages of 10 and 30. Osteosarcoma, on the other hand, can affect anyone at any age, including the elderly. Osteosarcoma affects around 10% of adults over the age of 60 [1]. 

The five-year survival rate indicates the percentage of children and teenagers who survive cancer for at least five years after diagnosis. A five-year survival rate of 68 percent has been reported for children with osteosarcoma aged 0 to 14. In addition, teenagers aged 15 to 19 have a five-year survival rate of 68%. If osteosarcoma is detected and treated before it spreads outside the site where it began, people of all ages have a 74% chance of survival for the next five years. Moreover, 66% of patients with bone cancer who survive five years have the cancer spread to surrounding tissue, organ, and lymph node systems. The chance of survival is 27% for the next five years, if the cancer has metastasized [2].

Osteosarcoma detection is a difficult and time-consuming task that demands a great deal of experience. Emerging new techniques and rapid processing technologies have led the health sector to adopt computerized diagnosis methods that can accurately predict whether a tumor is benign or malignant. The general design of these systems is a feature extractor followed by a classifier that takes the features as input. Some systems may also incorporate a pre-processing stage before the feature extractor, which is necessary to improve the image quality by cleaning the data input into the model. Noise elimination filters, contrast improvement, and other methods may be used in this pre-processing. The development of computer-assisted detection (CAD) systems [3,4,5,6,7] has been demonstrated by researchers as means for the segmentation and identification of osteosarcoma using various images, including computed tomography (CT) and magnetic resonance imaging (MRI) examinations. CT and MRI scans, however, have limitations. As a result, researchers are now using WSIs to identify osteosarcomas more accurately than CT scans and MRIs. Among the most common methods of identifying these cells is by staining tissue samples of the affected regions with hematoxylin and eosin [8]. The specimens are mounted on glass slides and then examined under a microscope by a pathologist after being stained. Whole slide images (WSIs) of good quality [9] were used in this paper; they are digital representations of glass slides without any pre-processing. 

Feature extraction is the next step in automated detection systems, and it can be performed manually or by deep learning (DL). Data are presented and understood better by reducing the dimensions of incoming data, known as feature extraction [10]. Handcrafted (HC) features are picture-specific properties determined by hand based on the targeted space’s characteristics, and these approaches are widely employed to extract them. Researchers have made extensive use of HC features as they are easy to extract, particularly in modest datasets. The characteristics can be determined with the help of professionals in the relevant sector. Due to their complexity, these features become difficult to determine when associated with complex images. In this case, deep learning models (DLMs) are used as a feature extraction technique. Due to recent improvements in the area of processing, such as the advent of quicker and more compact processors, DLMs have gained a great deal of traction in the previous decade, allowing professionals to quickly and easily train deeper networks. In this case, the DLM used for the extraction of features is usually a Convolutional Neural Network (CNN). These models can automatically learn attributes from an image, but for acceptable attribute derivation quality, they need a large training sample with a large amount of variance. Using them, one can better describe an image and enhance the feature analysis of it by providing more low-level characteristics. DLM has the advantage of not requiring any considerable pre-processing because it can perform the same operation automatically. However, the high computational and data requirements of such models are a drawback. 

## 2. Literature Review

Researchers have developed automatic detection systems for analyzing and classifying medical images (X-ray, ultrasound imaging, MRI, CT scan, histology images, etc.) to detect various types of malignancies and tumors as technology advances [11,12,13,14,15,16,17]. Several automated systems for categorizing medical images use handcrafted features [18,19,20,21], whereas other systems use DLMs to extract and classify medical images [22,23,24,25,26]. Recently, researchers have presented automatic detection systems [27,28] that combine both strategies, and these systems perform well when compared to their counterparts. The process of feature selection (FS) involves removing irrelevant features and redundant ones, thereby reducing the number of features. In the literature, researchers have presented a variety of metaheuristic algorithms to conduct FS [29,30,31,32,33]. El-Kenawy [34] diagnosed COVID-19 by using the Guided WOA algorithm, which is a method based on Stochastic Fractal Search. 

For the segmentation in CT and MRI images of osteosarcoma patients, many approaches have been suggested. Nasor and Obaid [35] advocated for employing a mixture of image processing techniques such as K-means clustering, Chan–Vese segmentation, iterative Gaussian filtering, and Canny edge detection to segment osteosarcomas in MRI images. The fundamental bone tumor has been studied by Vandana et al. [36]. They improved the graph cut-based clustering approach for distinguishing between malignant and healthy parts. Using multiclass irregular texture, they were able to measure the qualities of risk and categorize them as normal, benign, or malignant. 

In his study [37], Altameem performed segmentation in X-rays based on fuzzy ranking to diagnose bone tumors. Following segmentation, the various statistical characteristics are collected and treated using a deep neural network that applies the Levenberg–Marquardt learning algorithm. The inability of the MRI scans and CT scans to obtain cellular data limits the detectability of osteosarcoma using CAD systems. Contrary to MRIs and CT scans, WSIs can provide more detailed information about nuclei, including density and structure. Mishra et al. [38] reported low accuracy and inefficient models were used for classification. 

Arunachalam et al. [39] performed segmentation and extracted features from WSIs for the identification of osteosarcoma from a large dataset. Mishra et al. [40] suggested a CNN-based architecture as a means of classifying the dataset pictures into three categories: viable tumors, non-viable tumors, and non-tumors. They further fine-tuned and supplemented the CNN architecture by classifying a dataset of osteosarcoma WSIs and compared the findings to VGGNet, AlexNet, and LeNet. Due to training constraints, the picture size was decreased to 128 × 128 patches by cropping the original 1024 × 1024 image. The total accuracy was 92.4 percent, outperforming AlexNet, VGGNet, and LeNet. 

To categorize osteosarcoma WSIs, Arunachalam et al. [40] used a combination of ML and DLMs. K-means clustering was used as a segmentation method, followed by Otsu’s multi-level thresholding to extract ROIs from histology WSIs in the ML model. The pixels were clustered using a flood-fill approach, and the WSIs were then classified into viable tumors, non-viable tumors, and non-tumors by data analysis. Although the total number of ML models used for the segmentation of images was 13, support vector machine (SVM) provided the greatest accuracy. Their CNN approach was built on the foundations of AlexNet and LeNet. To overcome the size deficiency of the dataset, it was augmented using rotation and other techniques such as flipping. Images used for testing were converted into 128 × 128 patches and 1024 × 1024 tiles. In DLMs, SVM achieved accuracy of 89.9%, patches of 93.3 percent, and tiles of 91.2 percent.

D’Acunto et al. [41] applied deep learning approaches to classify human stromal cells as osteosarcoma cells. Their primary focus was on osteosarcoma. Their deep learning approach claimed 0.9715 ± 0.01 average accuracy. Anisuzzaman et al. [42] suggested a VGG19 and InceptionV3-based deep learning system that was trained via transfer learning. To increase the classification results, these classifiers were fed WSIs without patches as input. Due to memory constraints, the images needed to be reduced to 375 × 375 pixels. On multiclass classification, the cumulative accuracy of the VGG19 and InceptionV3 models was 93.91 percent and 78.26 percent, respectively.

The prior research has the following flaws:

- It used handcrafted features for tumor classification, which is a tedious task [17,18,19,20];
- MRI and CT scans were used for classification, which cannot provide cellular information [34,35,36];
- It reported low accuracy [38,40,43];
- There was no mechanism to ensure the security and privacy of patient data [35,36,37,38,39,40,41,42,43].

The following is an outline of this paper’s main contributions:

- We employed transfer learning with three different optimizers to minimize the training time and maximize the classification accuracy of the proposed model;
- The Internet of Medical Things (IoMT) has been incorporated into the proposed model for data collection;
- To ensure the security and privacy of the patient data, the proposed model uses blockchain technology;
- The suggested model employs edge computing to process and filter IoMT-generated data closer to the devices, resulting in increased speed and reliability;
- The fog computing layer is employed to further optimize the models and overcome the processing deficiency of the edge devices.

## 3. Materials and Methods

**Figure 1:**
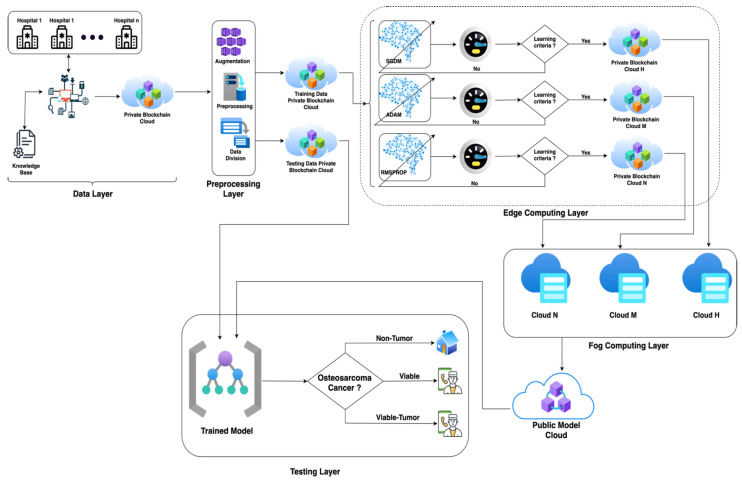
IoMT-based proposed model for the prediction of osteosarcoma cancer using transfer learning empowered with blockchain security, fog computing, and edge computing.

Figure 1 depicts the overall process for the prediction of osteosarcoma cancer using transfer learning empowered with blockchain security for patients’ data privacy and model security, fog computing, and edge computing to reduce the complexity of the problem. The proposed model consists of five layers: a data layer, pre-processing layer, edge computing layer, fog computing layer, and testing layer. First of all, the proposed model initiates the data layer and collects data using IoMT technology, and stores them in a blockchain-secured private data cloud. The data pre-processing layer imports raw data from the private cloud and applies numerous pre-processing techniques, including data augmentation, to compensate for the data deficiency, using different image parameters, such as image histogram equalization, for better training and testing results. The suggested methodology commences the data division process after the data pre-processing layer, dividing data into training and testing sets and storing pre-processed training data in the training data private blockchain cloud and testing data in the testing data private blockchain cloud. Following the data layer and pre-processing layer, the proposed model enters the edge computing layer to train the models and store them on edge clouds.

The edge computing layer imports training data from a private blockchain cloud and feeds them to the AlexNet algorithm along with stochastic gradient descent with momentum (SGDM), adaptive moment estimation (ADAM), and root mean squared propagation algorithms (RMSProp). The trained models with SGDM, ADAM, and RMSprop are stored in private blockchain cloud H, M, and N, respectively, if they meet the learning criteria; otherwise, the models are retrained from scratch. The fog computing layer is employed to further optimize the models and overcome the processing deficiency of the edge devices; based on the training accuracy, the best model is selected and stored in the public cloud. Following the diagnosis of osteosarcoma cancer, the patient may readily contact a specialist for early treatment and better medication to aid in the healing process.

The model’s performance and authenticity were verified using several statistical parameters, such as the Fowlkes–Mallows index (FMI), classification miss rate (CMR), positive predicted value (PPV), sensitivity (SEN), specificity (SPE), false negative rate (FNR), negative predicted value (NPV) F1 score, prediction accuracy (PA), false positive rate (FPR), likelihood positive ratio (LPR), and likelihood negative ratio (LNR).

(1) ωp=ϑpψp

∴ $\vartheta_{p}$ denotes the predicted class and $\psi_{p}$ the true class, and $\omega_{p}$ represents the true positive class.

(2) βp=∑h=13(ϑpψh≠p)

∴ $\beta_{p}\;\mathrm{represents}\;\mathrm{the}\;\mathrm{true}\;\mathrm{negative}\;\mathrm{class}$, the sum of all three predicted classes.

(3) ξp=∑h=13(ϑh≠pψp)

∴ $\xi_{p}\;\mathrm{represents}\;\mathrm{the}\;\mathrm{false}\;\mathrm{positive}\;\mathrm{class}$, the sum of all three predicted classes.

(4) γp=∑h=13(ϑh≠pψh≠p)

∴ $\gamma_{p}\;\mathrm{represents}\;\mathrm{the}\;\mathrm{false}\;\mathrm{negative}\;\mathrm{class},$ the sum of all three predicted classes.

(5) $$PA=\frac{\omega_{p}+{\;\beta }_{p}\;}{\omega_{p}+\beta_{p}+\xi_{p}+\gamma_{p}}*100$$

$∴\omega_{p}\;\mathrm{represents}\;\mathrm{the}\;\mathrm{true}\;\mathrm{positive}\;\mathrm{class},{\mathrm{and}\;\beta }_{p}\;\mathrm{represents}\;\mathrm{the}\;\mathrm{true}\;\mathrm{negative}\;\mathrm{class},\;\mathrm{while}$ $\xi_{p}\;\mathrm{represents}\;\mathrm{the}\;\mathrm{false}\;\mathrm{positive}\;\mathrm{class}$, and $\gamma_{p}\;\mathrm{represents}\;\mathrm{the}\;\mathrm{false}\;\mathrm{negative}\;\mathrm{class}.$

(6) $$\mathrm{CMR}=100-\left(\frac{\omega_{p}+{\;\beta }_{p}\;}{\omega_{p}+{\;\beta }_{p}+\xi_{p}+\gamma_{p}}*100\right)$$

(7) $$\mathrm{SEN}=\frac{\omega_{p}}{\omega_{p}+\gamma_{p}}\;*\;100\;$$

(8) $$\mathrm{SPE}=\frac{\beta_{p}}{\beta_{p}+\xi_{p}}\;*\;100\;$$

(9) $$F1\text{-}\mathrm{Score}=\frac{2{\;\omega }_{p}}{2{\;\omega }_{p}+\xi_{p}+\gamma_{p}}*100$$

(10) $$\mathrm{PPV}=\frac{\omega_{p}}{\omega_{p}+\xi_{p}}*100$$

(11) $$\mathrm{NPV}=\frac{\beta_{p}}{\beta_{p}+\gamma_{p}}*100$$

(12) $$\mathrm{FPR}=100-\left(\frac{\beta_{p}}{\beta_{p}+\xi_{p}}*100\right)$$

(13) $$\mathrm{FNR}=100-\left(\frac{\omega_{p}}{\omega_{p}+\gamma_{p}}*100\right)$$

(14) $$\mathrm{LPR}=\frac{\frac{\omega_{p}}{\omega_{p}+\gamma_{p}}*100}{100-\left(\frac{\beta_{p}}{\beta_{p}+\xi_{p}}*100\right)}$$

(15) $$\mathrm{LNR}=\frac{100-\left(\frac{\omega_{p}}{\omega_{p}+\gamma_{p}}*100\right)}{\frac{\beta_{p}}{\beta_{p}+\xi_{p}}*100}$$

(16) $$\mathrm{FMI}=\sqrt{\left(\frac{\omega_{p}}{\omega_{p}+\gamma_{p}}*100\right)*\left(\frac{\omega_{p}}{\omega_{p}+\xi_{p}}*100\right)}$$

In Table 1, the overall process is illustrated step by step in pseudocode form. The proposed pseudocode shows the overall approach to predicting the osteosarcoma using the transfer learning approach. Each step describes how the model fetches data, pre-processes them, uses the blockchain cloud for patient data privacy and cyber security, and explains edge computing and fog computing to implement the model. Finally, the pseudocode describes the testing phase and statistical parameter calculation. The proposed pseudocode covers all limitations of previous studies. 

## 4. Dataset

**Figure 2:**
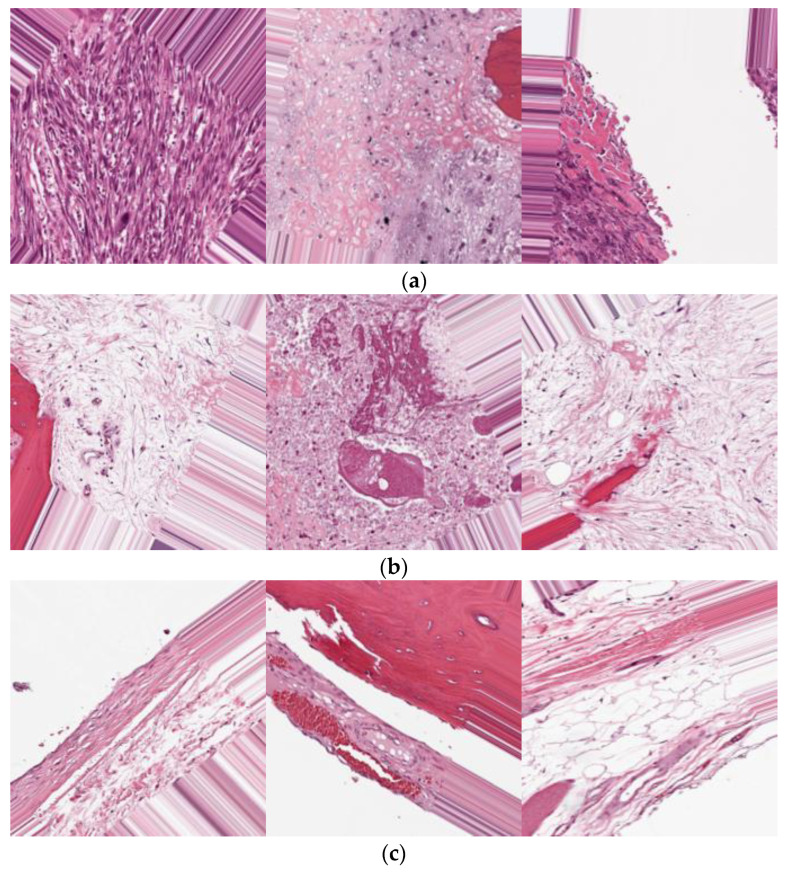
Data samples from each prediction class: (**a**) viable, (**b**) viable tumor, (**c**) non-tumor.

Hematoxylin and eosin (H&E)-stained osteosarcoma histology images composed the dataset used in this study [9]. A team of clinical investigators from the University of Texas Southwestern Medical Center in Dallas gathered the data. This dataset was created from archival samples from 50 patients treated at the Children’s Medical Center in Dallas between 1995 and 2015. According to the prevalent cancer type in each image, the photos were labeled as non-tumor, viable tumor, or viable. Two medical specialists worked on the annotation. The dataset contains 1144 photos with a resolution of 1024 × 1024, with the following distribution: 536 (47%) non-tumor images, 263 (23%) necrotic tumor images, and 345 (30%) viable tumor tiles. As a publicly available dataset, it contains 1144 photos of three classes, which was not enough for the training and testing process. Thus, to balance the dataset, data augmentation techniques were applied in such a way that each class would contain 1100 images. For transfer learning, the proposed pseudocode applies a pre-processing technique to set the input image resolution to 227 × 227 pixels. Some samples from each prediction class are presented in Figure 2.

## 5. Simulation and Results

The suggested model in this work utilized IoMT-based transfer learning with data and model security provided by a blockchain. For the training and testing of the proposed model, we used a MacBook Pro 2017, 16 GB RAM, 512 GB SSD with integrated GPU. The proposed model splits patients’ data into 70% and 30% for training and testing, respectively. To measure the performance of the transfer learning model empowered with blockchain, edge computing, and fog computing, numerous statistical parameters have been used.

**Figure 3:**
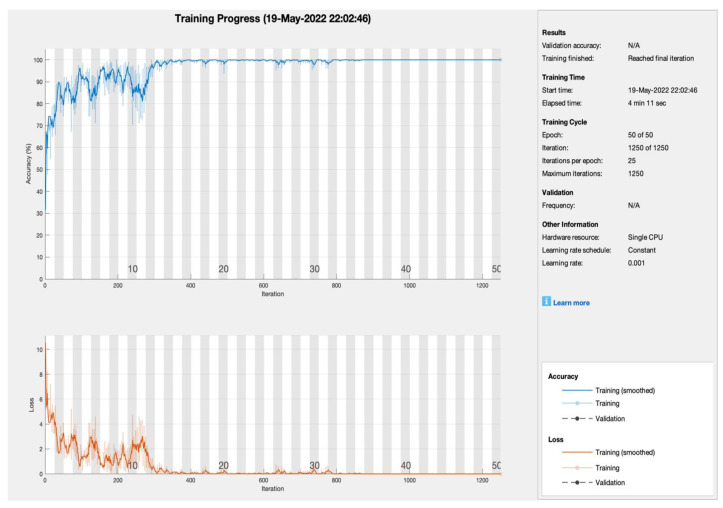
Training progress of SGDM empowered with blockchain, edge computing, and fog computing.

The proposed model applies various combinations of iterations, epochs, and learning rate, so, in this study, the proposed approach was applied to obtain the best hit results in order to demonstrate its performance further. Figure 3 shows the training progress of the proposed model utilizing SGDM with blockchain security, edge computing, and fog computing. To train the model, the suggested model employed 50 epochs, 1250 iterations, and 25 iterations per epoch with a single CPU and a learning rate of 0.001. The model converged after the tenth epoch and remained stable until the 50th epoch. As a result, the suggested model with the SGDM learner obtained PA and CMR of 99.8% and 0.2%, respectively.

**Figure 4:**
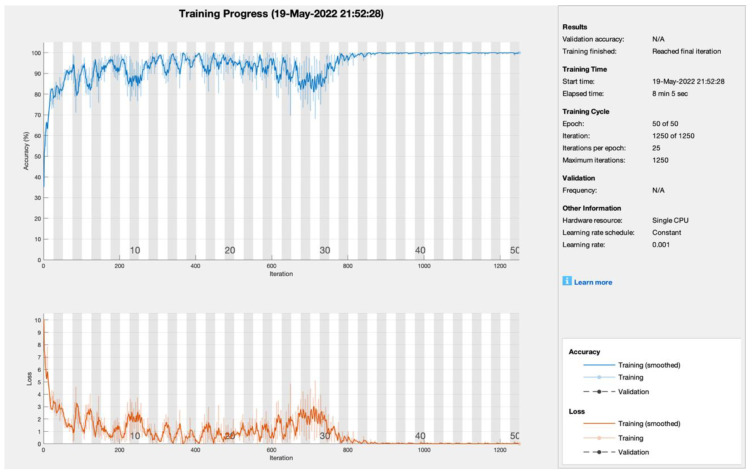
Training progress of ADAM empowered with blockchain, edge computing, and fog computing.

**Figure 5:**
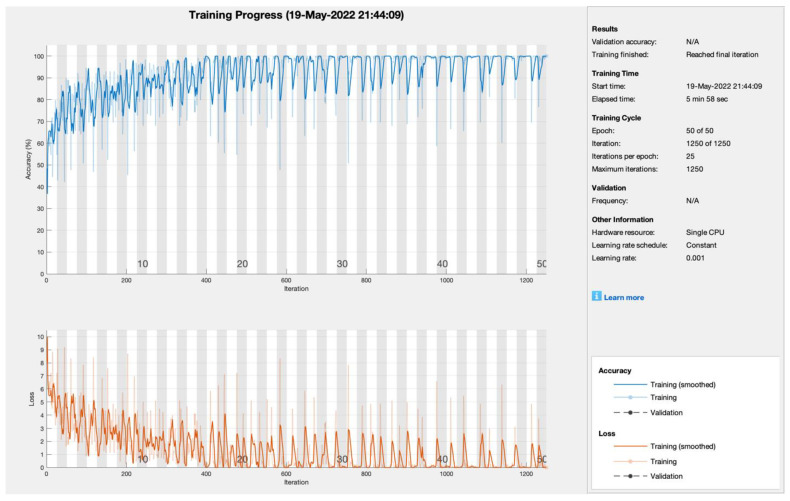
Training progress of RMSProp empowered with blockchain, edge computing, and fog computing.

Figure 4 illustrates the training progress of the proposed model with ADAM. The training was conducted on a single CPU with a 0.001 learning rate, 50 epochs, 1250 iterations, and 25 iterations per epoch. The model reached convergence at the 30th epoch and remained stable until the 50th epoch. As a result, the suggested model with ADAM obtained 99.5 percent PA and 0.5 percent CMR. The suggested model with the RMSProp learner was trained using 50 epochs, 1250 iterations, and 25 iterations per epoch with a single CPU with a learning rate of 0.001. The model did not converge and had random training fluctuations until the 50th epoch, which was not helpful for prediction, as shown in Figure 5. However, the suggested model with RMSProp achieved 99.5 percent PA and 0.5 percent CMR.

Table 2 displays the total comparative training outcomes for all learners, revealing that SGDM outperformed all others, achieving 99.8% PA and 0.2% CMR, respectively. ADAM obtained 99.5% PA and 0.5% CMR, respectively. Finally, RMSProp obtained 99.5% and 0.5% PA and CMR, respectively, but its findings were inconsistent, which is not favorable for prediction procedures. As Table 2 further depicts the simulation results, it shows that the proposed model applied 20 and 30 epochs to obtain better results, with 25 iterations per epoch; as the number of epochs decreases, the results become worse because the dataset is larger, so the proposed model achieved the best accuracy results on 50 epochs, which were far better than the results obtained with 20 and 30 epochs. Overall, the simulation illustrates that as the iterations increase, the prediction results become better. 

The SGDM confusion matrix is shown in Table 3. In 987 instances, the suggested approach successfully identified non-tumor slides, with only three exceptions. In the instance of viable tumors, 987 forecasts were correct, while three were erroneous. There were 984 valid viable predictions, whereas six were incorrect. Table 4 shows the testing confusion matrix of the proposed model with ADAM. The suggested model accurately predicted 985 non-tumors, 985 viable tumors, and 980 viable cases, whereas it incorrectly predicted 5 non-tumors, 5 viable tumors, and 10 non-viable tumors.

The proposed model accurately predicted 987 non-tumor, 986 viable tumor, and 983 viable cases, while 3 non-tumor, 4 viable tumor, and 7 non-viable tumor cases were incorrectly predicted, as shown in Table 5. The overall statistical performance of the proposed model is shown in Table 6. The proposed model with SGDM achieved 99.30%, 0.70%, 98.80%, 99.55%, 98.95%, 99.10%, 99.40%, 0.45%, 1.20%, 218.02, 0.01% and 98.95% of PA, CMR, Sen, Spec, F1, PPV, NPV, FPR, FNR, LPR, LNR and FMI, respectively. With ADAM, the model achieved 99.09% PA, 0.91% CMR, 98.20% Sen, 99.55% Spec, 98.65% F1, 99.09% PPV, 99.10% NPV, 0.45% FPR, 1.80% FNR, 216.05 LPR, 0.02% LNR and 98.65% FMI. RMSProp obtained 99.30%, 0.70%, 98.80%, 99.55%, 98.95%, 99.10%, 99.40%, 0.45%, 1.20%, 218.02, 0.01% and 98.95% of PA, CMR, Sen, Spec, F1, PPV, NPV, FPR, FNR, LPR, LNR and FMI, respectively. As seen in the table, SGDM and RMSProp obtained almost identical outcomes; however, RMSProp’s training progress showed numerous fluctuations, making the RMSProp findings ineffective.

We present a comparison of the proposed model with state-of-the-art models in Table 7. It is evident from the table that our proposed methodology has outperformed all current models for osteosarcoma detection in terms of accuracy. The studies cited in [38,40,41] reported low accuracy and inefficient models were used for classification. Although a few other studies reported promising results [39,43,44], they are unable to ensure the security of patient data, as well as of the trained model. Our model achieved very high accuracy of up to 99.30%. Moreover, our model uses blockchain for the security of data as well as the trained model, while edge computing and fog computing facilitate the faster and more reliable processing of IoMT-generated data. 

## 6. Conclusions and Future Work

This paper presents a novel model for bone cancer classification, which is not only accurate in the detection and classification of the disease but also ensures the privacy and integrity of patient data by implementing blockchain, fog computing, and edge computing. Transfer learning has been employed in our model to reduce the model’s training time. We employed three different optimization algorithms for training, namely SGDM, ADAM, and RMSprop. The highest training and testing accuracy was 99.30%, achieved by SGDM, followed by 99.20% for RMSprop and 99.09% for ADAM. In the future, we intend to investigate new deep learning models that are both computationally and performance-wise superior to the existing models. This might assist in lessening the computing burden and speeding up the system even further. 

## Figures and Tables

**Table 1:** Pseudocode for the suggested model for osteosarcoma cancer prediction utilizing transfer learning and IoMT with blockchain security, edge computing, and fog computing.

Steps	Code
1	Data source (hospital_1_, hospital_2_, hospital_3_, ………, hospital) (knowledge base)
2	IoMT (data source)
3	Private cloud blockchain secured (data)
4	Data pre-processing (augmentation, contrast correction, data division)
5	Store pre-processed train data→private cloud (blockchain secured)
6	Store pre-processed test data→private cloud (blockchain secured)
7	Edge computing layer Transfer learning SGDM→private cloud H (blockchain secured) ADAM→private cloud M (blockchain secured) RMSPROP→Private Cloud N (blockchain secured) Fog computing layer Cloud N← Cloud M← Cloud H← Public model cloud (trained model)←
8	Import test data→private cloud Import trained model→public model cloud
9	Apply testing (predict osteosarcoma cancer)
10	Apply statistical matrix (model performance)

**Table 2:** Training results of AlexNet models with numerous learners.

AlexNet					
Model	Iterations	Learning Rate	Epoch	PA (%)	CMR (%)
**SGDM**	1250	0.001	50	99.8	0.2
**ADAM**				99.5	0.5
**RMSProp**				99.5	0.5
**SGDM**	750	0.001	30	98.88	1.12
**ADAM**				98.01	1.99
**RMSProp**				97.99	2.01
**SGDM**	500	0.001	20	92.8	7.2
**ADAM**				91.5	8.5
**RMSProp**				90.06	9.94

**Table 3:** Testing confusion matrix of SGDM empowered with transfer learning.

Total Samples (990)	Non-Tumor	Viable Tumor	Viable
**Non-Tumor**	328	0	3
**Viable Tumor**	0	330	5
**Viable**	2	0	322

**Table 4:** Testing confusion matrix of ADAM empowered with transfer learning.

Total Samples (990)	Non-Tumor	Viable Tumor	Viable
**Non-Tumor**	330	0	3
**Viable Tumor**	0	330	4
**Viable**	0	0	323

**Table 5:** Testing confusion matrix of RMSProp empowered with transfer learning.

Total Samples (990)	Non-Tumor	Viable Tumor	Viable
**Non-Tumor**	330	0	3
**Viable Tumor**	0	329	2
**Viable**	0	1	325

**Table 6:** Testing statistical parameter results of the proposed model empowered with transfer learning.

Solver Name	Statistical Parameters											
	PA	CMR	Sen	Spec	F1	PPV	NPV	FPR	FNR	LPR	LNR	FMI
**SGDM**	99.30	0.70	98.80	99.55	98.95	99.10	99.40	0.45	1.20	218.02	0.01	98.95
**ADAM**	99.09	0.91	98.20	99.55	98.65	99.09	99.10	0.45	1.80	216.05	0.02	98.65
**RMSPROP**	99.30	0.70	98.80	99.55	98.95	99.10	99.39	0.45	1.20	217.37	0.01	98.95

**Table 7:** Comparison of the proposed model with state-of-the-art models.

Article Authors	Year	Model/Classifier	Blockchain	IoMT	Fog/Edge Computing	Accuracy (%)
Mishra, Rashika et al. [38]	2017	CNN	No	No	No	84
Arunachalam, Harish Babu et al. [39]	2017	K-means, flood-fill algorithm	No	No	No	95.5
Mishra et al. [40]	2018	AlexNet, LeNet, and VGGNet	No	No	No	92
Anisuzzaman et al. [42]	2021	VGG19	No	No	No	96
Arunachalam, Harish Babu et al. [43]	2019	Complex trees (CT) Support vector Machine (SVM) Ensemble learners (ENS)	No	No	No	89.9
Liangrui Pan et.al. [44]	2022	NRCA-FCFL	No	No	No	99.17
**Proposed Model**	**2022**	**AlexNet with SGDM, ADAM, RMSprop. Blockchain, edge, and fog computing**	**Yes**	**Yes**	**Yes**	**99.30**

## Data Availability

The simulation files/data used to support the findings of this study are available from the corresponding author upon request.

## References

[1] American Cancer Society. https://www.cancer.org/cancer/osteosarcoma/about/key-statistics.html#:~:text=Osteosarcoma%20is%20not%20a%20common,ages%20of%2010%20and%2030.

[2] Seigel R.L., Miller K.D., Fuchs H.E., Jemal A. (2022). Cancer Statistics 2022. CA A Cancer J. Clin..

[3] Chen C.X., Zhang D., Li N., Qian X.J., Wu S.J., Gail S. (2013). Osteosarcoma segmentation in MRI based on Zernike moment and SVM. Chin. J. Biomed. Eng..

[4] Jia H., Zhao X., Qin L., Cai X. (2021). Imaging method for osteosarcoma diagnosis and clinical staging information optimization. J. Med. Imaging Health Inform..

[5] Baidya Kayal E., Kandasamy D., Sharma R., Bakhshi S., Mehndiratta A. (2019). Segmentation of osteosarcoma tumor using diffusion weighted MRI: A comparative study using nine segmentation algorithms. Signal Image Video Process..

[6] Loraksa C., Mongkolsomlit S., Nimsuk N., Uscharapong M., Kiatisevi P. (2022). Effectiveness of Learning Systems from Common Image File Types to Detect Osteosarcoma Based on Convolutional Neural Networks (CNNs) Models. J. Imaging.

[7] Zhang R., Huang L., Xia W., Zhang B., Qiu B., Gao X. (2018). Multiple supervised residual network for osteosarcoma segmentation in CT images. Comput. Med. Imaging Graph..

[8] Goode A., Gilbert B., Harkes J., Jukic D., Satyanarayanan M. (2013). OpenSlide: A vendor-neutral software foundation for digital pathology. J. Pathol. Inform..

[9] Leavey P., Sengupta A., Rakheja D., Daescu O., Arunachalam H.B., Mishra R. (2019). Osteosarcoma data from UT Southwestern/UT Dallas for Viable and Necrotic Tumor Assessment. Cancer Imaging Arch..

[10] Ding C., He X., Zha H., Simon H.D. (2002). Adaptive dimension reduction for clustering high dimensional data. Proc. Int. Conf. Data Min..

[11] Mehmood S., Ghazal T.M., Khan M.A., Zubair M., Naseem M.T., Faiz T., Ahmad M. (2022). Malignancy Detection in Lung and Colon Histopathology Images Using Transfer Learning with Class Selective Image Processing. IEEE Access.

[12] Taleb N., Mehmood S., Zubair M., Naseer I., Mago B., Nasir M.U. Ovary Cancer Diagnosing Empowered with Machine Learning. Proceedings of the 2022 International Conference on Business Analytics for Technology and Security (ICBATS).

[13] Nadeem M.W., Goh H.G., Khan M.A., Hussain M., Mushtaq M.F., Ponnusamy V.A. (2021). Fusion-based machine learning architecture for heart disease prediction. Comput. Mater. Contin..

[14] Siddiqui S.Y., Athar A., Khan M.A., Abbas S., Saeed Y., Khan M.F., Hussain M. (2021). Modelling, simulation, and optimization of diagnosis cardiovascular disease using computational intelligence approaches. J. Med. Imaging Health Inform..

[15] Ahmed U. (2022). Prediction of Diabetes Empowered with Fused Machine Learning. IEEE Access.

[16] Nasir M.U., Khan M.A., Zubair M., Ghazal T.M., Said R.A., Al Hamadi H. (2022). Single and mitochondrial gene inheritance disorder prediction using machine learning. Comput. Mater. Contin..

[17] Rahman A.U., Alqahtani A., Aldhafferi N., Nasir M.U., Khan M.F., Khan M.A., Mosavi A. (2022). Histopathologic Oral Cancer Prediction Using Oral Squamous Cell Carcinoma Biopsy Empowered with Transfer Learning. Sensors.

[18] Bakheet S. (2017). An svm framework for malignant melanoma detection based on optimized hog features. Computation.

[19] Khan M.Q., Hussain A., Rehman S.U., Khan U., Maqsood M., Mehmood K., Khan M.A. (2019). Classification of melanoma and nevus in digital images for diagnosis of skin cancer. IEEE Access.

[20] Rahmawaty M., Nugroho H.A., Triyani Y., Ardiyanto I., Soesanti I. Classification of breast ultrasound images based on texture analysis. Proceedings of the 2016 1st International Conference on Biomedical Engineering (IBIOMED).

[21] Solmaz A., Tajeripour F. (2016). Detection of brain tumor in 3D MRI images using local binary patterns and histogram orientation gradient. Neurocomputing.

[22] Bansal P., Kumar S., Srivastava R., Agarwal S. (2021). Using transfer learning and hierarchical classifier to diagnose melanoma from dermoscopic images. Int. J. Healthc. Inf. Syst. Inform. (IJHISI).

[23] Bisla D., Choromanska A., Berman R.S., Stein J.A., Polsky D. Towards automated melanoma detection with deep learning: Data purification and augmentation. Proceedings of the IEEE/CVF Conference on Computer Vision and Pattern Recognition Workshops.

[24] Cao Z., Duan L., Yang G. (2019). An experimental study on breast lesion detection and classification from ultrasound images using deep learning architectures. BMC Med. Imaging.

[25] Li Y., Deng L., Yang X. (2019). Early diagnosis of gastric cancer based on deep learning combined with the spectral-spatial classification method. Biomed. Opt. Express.

[26] Srinivasu P.N., SivaSai J.G., Ijaz M.F., Bhoi A.K., Kim W., Kang J.J. (2021). Classification of skin disease using deep learning neural networks with MobileNet V2 and LSTM. Sensors.

[27] Almaraz-Damian J.-A., Ponomaryov V., Sadovnychiy S., Castillejos-Fernandez H. (2020). Melanoma and nevus skin lesion classification using handcraft and deep learning feature fusion via mutual information measures. Entropy.

[28] Hasan A.M., Jalab H.A., Meziane F., Kahtan H., Al-Ahmad A.S. (2019). Combining deep and handcrafted image features for MRI brain scan classification. IEEE Access.

[29] Abdel-Basset M., El-Shahat D., El-henawy I., de Albuquerque V.H.C., Mirjalili S. (2020). A new fusion of grey wolf optimizer algorithm with a two-phase mutation for feature selection. Expert Syst. Appl..

[30] Al-Tashi Q., Abdulkadir S.J., Rais H.M., Mirjalili S., Alhussian H., Ragab M.G., Alqushaibi A. (2020). Binary multi-objective grey wolf optimizer for feature selection in classification. IEEE Access.

[31] Bansal P., Kumar S., Pasrija S., Kumar S. (2020). A hybrid grasshopper and new cat swarm optimization algorithm for feature selection and optimization of multi-layer perceptron. Soft Comput..

[32] Elgamal Z.M., Yasin N.B.M., Tubishat M., Alswaitti M., Mirjalili S. (2020). An improved harris hawks optimization algorithm with simulated annealing for feature selection in the medical field. IEEE Access.

[33] El-Kenawy E.M., Eid M.M., Saber M., Ibrahim A. (2020). MbGWO-SFS: Modified binary grey wolf optimizer based on stochastic fractal search for feature selection. IEEE Access.

[34] El-Kenawy E.M., Ibrahim A., Mirjalili S., Eid M.M., Hussein S.E. (2020). Novel feature selection and voting classifier algorithms for COVID-19 classification in CT images. IEEE Access.

[35] Nasor M., Obaid W. (2021). Segmentation of osteosarcoma in MRI images by K-means clustering, Chan-Vese segmentation, and iterative Gaussian filtering. IET Image Process..

[36] Vandana B.S., Antony P.J., Alva S.R. (2020). Analysis of malignancy using enhanced graphcut-based clustering for diagnosis of bone cancer. Information and Communication Technology for Sustainable Development.

[37] Altameem T. (2020). Fuzzy rank correlation-based segmentation method and deep neural network for bone cancer identification. Neural Comput. Appl..

[38] Mishra R., Daescu O., Leavey P., Rakheja D., Sengupta A. (2017). Histopathological diagnosis for viable and non-viable tumor prediction for osteosarcoma using convolutional neural network. Proceedings of the 13th International Symposium on Bioinformatics Research and Applications (ISBRA).

[39] Arunachalam H.B., Mishra R., Armaselu B., Daescu O., Martinez M., Leavey P., Rakheja D., Cederberg K., Sengupta A., Ni’Suilleabhain M. Computer aided image segmentation and classification for viable and non-viable tumor identification in osteosarcoma. Proceedings of the Pacific Symposium on Biocomputing.

[40] Mishra R., Daescu O., Leavey P., Rakheja D., Sengupta A. (2018). Convolutional neural network for histopathological analysis of osteosarcoma. J. Comput. Biol..

[41] D’Acunto M., Martinelli M., Morono D. (2020). From human mesenchymal stromal cells to osteosarcoma cells classification by deep learning. J. Intell. Fuzzy Syst..

[42] Anisuzzaman D.M., Barzekar H., Tong L., Luo J., Yu Z. (2020). A deep learning study on osteosarcoma detection from histological images. arXiv.

[43] Arunachalam H.B., Mishra R., Daescu O., Cederberg K., Rakheja D., Sengupta A., Leonard D., Hallac R., Leavey P. (2019). Viable and necrotic tumor assessment from whole slide images of osteosarcoma using machine-learning and deep-learning models. PLoS ONE.

[44] Pan L., Wang H., Wang L., Ji B., Liu M., Chongcheawchamnan M., Yuan J., Peng S. (2022). Noise-reducing attention cross fusion learning transformer for histological image classification of osteosarcoma. Biomed. Signal. Process. Control..

